# Electrochemical study of anti-platelets tirofiban HCl in dosage form using nanomaterials modified sensors: developed and green assessed by eco-scale and complex-GAPI approach

**DOI:** 10.1186/s13065-024-01358-1

**Published:** 2025-01-08

**Authors:** Mohamed. Rizk, Maha Mahmoud Abou El-Alamin, Ola Abd Elkhalek, Hassan A. M. Hendawy

**Affiliations:** 1 Department of Pharmaceutical Analytical Chemistry, Faculty of Pharmacy, Helwan University, P.O.Box 11795, Cairo, Egypt; 2 National Organization for Drug Control and Research (NODCAR), P.O.Box 29, Cairo, Egypt

**Keywords:** Carbon paste modifications, Eco-friendly, Electrochemical analysis, Tirofiban, Antiplatelet

## Abstract

Tirofiban hydrochloride is used to inhibit platelet aggregation, which has a significant impact on the treatment of congestive heart failure the most common cause of death according to WHO. Therefore, its quantification in pharmaceutical dosage form is critical. In this work, an electrochemical method for the determination of tirofiban HCl in pharmaceutical dosage form was developed and validated. Carbon paste electrode modified with multi-walled carbon nanotubes (MWCNT) was utilized to examine the electrochemical response of tirofiban hydrochloride. Scanning electron microscopy and Energy-dispersive X-ray analysis was used to investigate the morphology of this electrode. A linear response was obtained within the range of (27.00–745.00 ng/mL) (5.4 × 10^–5^ M–1.5 × 10^−3^ M) with a correlation coefficient of 0.9995, and a detection limit of 15.50 ng*/*mL (3.1 × 10^–5^ M). The greenness profile of the method was assessed utilizing the eco-scale and the green analytical procedure Index.

## Introduction

Tirofiban hydrochloride (TIR) is an antiplatelet drug that acts as a reversible antagonist of the platelet glycoprotein IIb/IIIa receptor, which is known as a therapeutic target in decreasing the development of platelet-dependent thrombus [1]. Therefore, it is used to treat heart attacks by preventing blood clots in people who have severe chest pain and in those who are undergoing an angioplasty procedure to open blocked arteries [2].

**Fig. 1:**
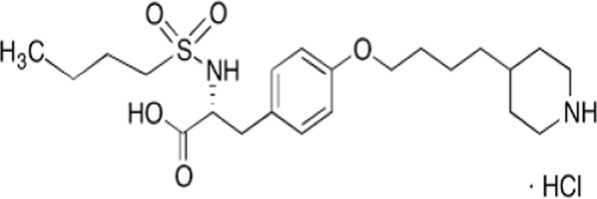
Chemical structure of tirofiban HCl

TIR is defined chemically as N-(butylsulfonyl)-*O*-(4-[4-piperidinyl]butyl)-L-tyrosine monohydrochloride monohydrate Fig. 1. It was approved by FDA in 1998. It was marketed in the United States as Aggrastat^®^. From the literature, it was found the drug was determined by the HPLC method [3–10], TLC and spectrophotometric method [10], also LC–MS-MS [11–15], and spectrofluorimetric method [16]. A literature survey revealed that there was one electrochemical method has been reported for the estimation of TIR in dosage form [17]. This work aims to estimate TIR by an electroanalytical method which unlike other analytical methods such as HPLC that expensive method, requires a complicated pretreatment process, requires a large number of expensive organic solvents, and needs high power supply [18], UV spectrophotometry which is unselective and LC–MS despite its high sensitivity, it is generally highly operational cost. Electroanalytical methods are attracting much attention because of their advantage over conventional methods, such as low-cost instrumentation, speed, simplicity, sensitivity, and providing information about the drug mechanism [19]. Carbon paste electrodes (CPEs) have been developed rapidly due to their stable response, and eco-friendly. They provide an easily renewable surface for electron exchange. Modification of CPEs with different nanomaterials increases CPEs sensitivity and reproducibility due to their role in increasing the surface area and improving electron transfer [20]. Herein we tried different types of nanoparticles as multi-wall carbon nanotube (MWCNT), graphene (Gr), zinc oxide (ZnO), and iron oxide (Fe_2_O_3_).

## Experimental

### Materials and reagents

TIR active constituent TIR and Clograstat^®^12.5 mg/50 mL I.V infusion were kindly supplied by Global pharmaceutical industry, 6th of October City, Egypt. Gr powder, MWCNT (MWCNTs, > 95% carbon, multi-walled O.D x L 6–9 nm × 5 µm, ZnO, Fe_2_O_3_, paraffin oil, phosphoric acid, boric acid, glacial acetic acid, hydrochloric acid, graphite powder, potassium biphthalate, sodium hydroxide, ascorbic acid, mannitol, lactose, sodium chloride, and methanol were taken from (Sigma-Aldrich, Germany) 0.0.04 M Britton Robinson buffer (BR) solutions of pH 2.0–12.0 and acetate buffer of pH 4.0 were prepared according to US Pharmacopeia[21]. Solvents and reagents utilized during the procedure were of analytical grade. All experiments were performed at room temperature.

### Apparatus

Voltammetric measurements were made with 884 professional VA Metrohm voltameter a data was analyzed with viva version 2.0 software. The 3 electrodes system consists of bare or modified CPE as a working electrode, Ag/AgCl (3 mol/L KCl) as a reference electrode, and platinum wire as the auxiliary electrode. A glass cell (50 mL) was utilized for electrochemical measurements, pH measurements were done by Hanna pH meter (HI 2211 PH/ORP) and Vortex mixer, USA. All electrochemical experiments were held at room temperature.

### Working electrode preparation

Simply CPE was formulated by mixing well 500 mg graphite powder with 0.2 mL of paraffin oil using a mortar and pestle then packing the uniform paste into an electrode cavity. The outer surface of the electrode was polished on filter paper till it gave a glowing look and connecting it to electricity with a copper wire. Nano-modified electrodes ZnO-NPs/CPE-Fe_2_O_3_-NPs/CPE-Gr/CPE-MWCNT/CPE were prepared by replacing 5% of graphite powder with different nanomaterial, and the paste was prepared by the same manner.

### Standard solutions

Stock standard solution of TIR was prepared by dissolving 10.0 mg of TIR in a 10 mL volumetric flask with methanol this solution is utilized for the preparation of more dilute solutions and kept in a refrigerator at 4 ºC. The working solution was prepared by diluting 1.0 mL of stock standard solution in a 100 mL volumetric flask using double distilled water.

### Procedure of calibration curve

Appropriate aliquots of working solution of TIR were transferred to 10 mL volumetric flasks then the solutions were completed by 0.04 M BR-buffer pH 4.0 to the mark to cover the linear concentration range of TIR. Differential pulse voltammetry (DPV) was recorded at a scan rate 100 mV/s. The calibration curve was recorded by plotting the peak current against the TIR concentration in (ng/mL).

### Application

#### Preparation of pharmaceutical dosage form solution

Clograstat^®^ (12.5 mg /50 mL) IV,1 mL of this solution was transferred to a 10 mL volumetric flask using methanol then different aliquots of this solution were added to a 10 mL volumetric flask and the volume was completed with BR-buffer at pH 4.0 to the mark and analyzed using the method described above. A standard addition technique was carried out for the determination of TIR in its dosage form. Known concentrations of TIR standard solution was added to the solution containing Clograstat^®^ IV and then quantitatively measured.

## Results and discussion

### Optimization of the method parameters

#### Influence of buffer PH and buffer type

To acquire the most favorable experimental conditions, the influence of pH values on the electrochemical behavior of TIR was examined by DPV in 0.04 M BR buffer over the pH range (2.0–12.0) on a bare CPE.

**Fig. 2:**
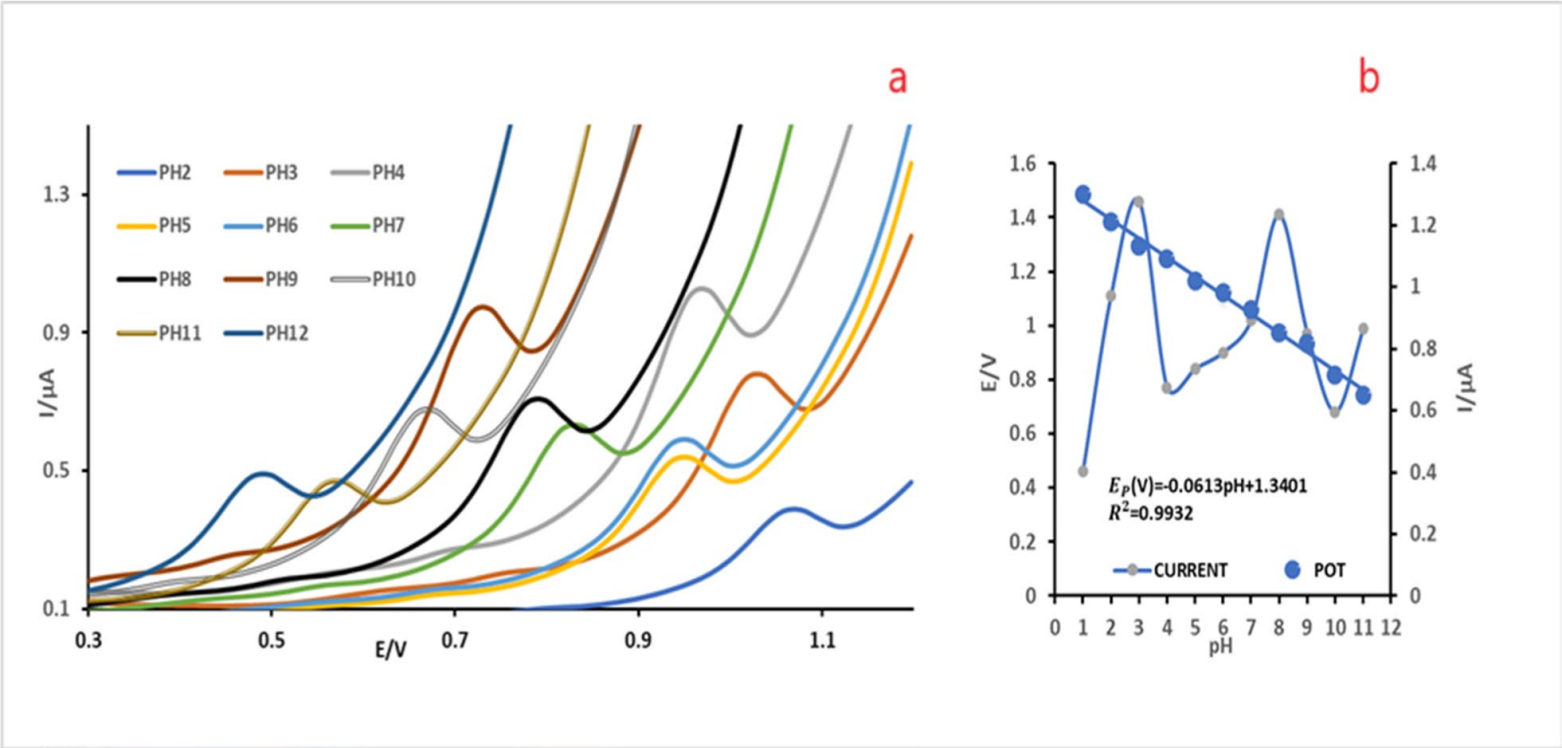
**a** DP voltammogram of 198.0 ng/mL (3.9 × 10^–4^ M) of TIR in 0.04 M BR buffer of different pH values (2.0–12.0) at bare CPE. **b** Plot of peak potential and peak current against pH (2.0–12.0) of 198.0 ng/mL (3.9 × 10^–4^ M) of TIR in 0.04 M BR buffer at bare CPE

As shown in Fig. 2a the plot of peak current (Ip) vs pH revealed that pH 4.0 had the highest peak current value, so it was chosen as the optimal pH. Figure 2b shows the relationship between pH and peak potentials (Ep). The anodic peak potential decreased with increasing pH, indicating that TIR showing oxidation was pH-dependent involving protonation/deprotonation in the charge transfer procedure. This relation between pH vs Ep resulted in a straight line with a slope value of (**− **0.0613 mV^−1^). As stated by the following equations, **(V) = − 0.0613pH + 1.3401** the anodic peak potential varied linearly with the pH Slope value indicating that the number of electrons is equivalent to the number of protons in the electro-oxidation reaction [22].

**Fig. 3:**
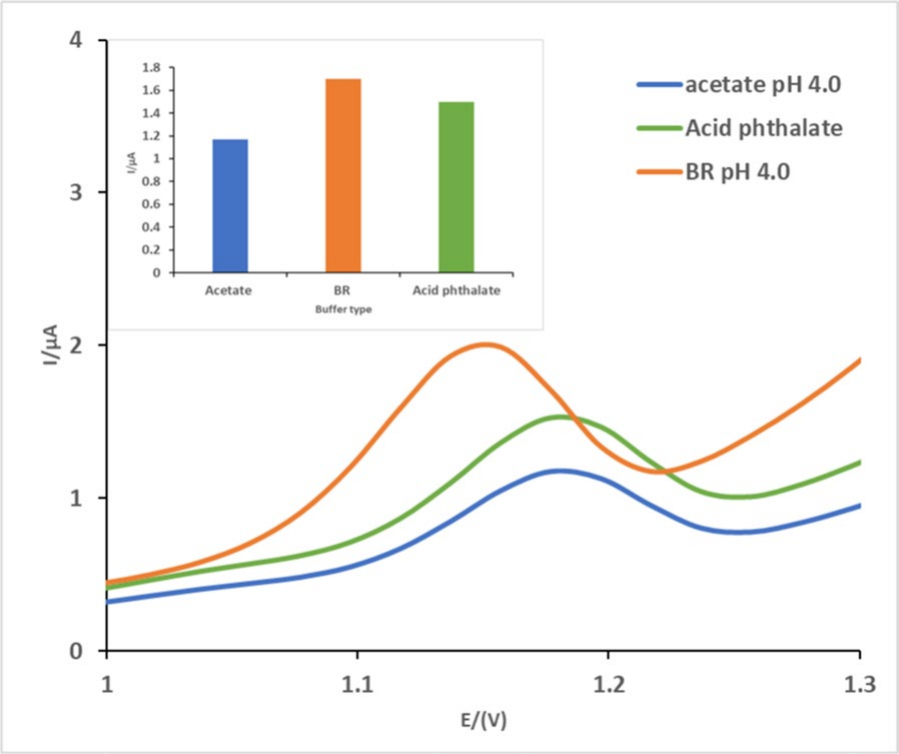
DP voltammogram of 198.0 ng/mL (3.9 × 10^–4^ M) of TIR in different buffer types (BR-acetate- acid phthalate) pH 4.0 at bare CPE. The inset: plot of anodic peak current against different buffer types

For further measurements, acetate and acid phthalate buffers were used as another supporting electrolyte solution at this best pH (4.0). As shown in Fig. 3 BR buffer was better as it showed the highest current and the best peak appearance.0.04 M BR buffer of pH 4.0 was selected as the best electrolyte buffer solution to utilize in the following procedure.

#### Electrooxidation on the modified electrode

The selection of an appropriate electrode is a critical step in developing an efficient voltammetric assay. Nanostructures with their distinct characteristics are regarded as a promising approach for improving sensor performance. So, electrodes were prepared to utilize MWCNTs, Gr-NPs, ZnO-NPs, and Fe_2_O_3_-NPs. The performance of partial carbon replacement with 5% nanomaterials varied depending on the nanomaterial.

**Fig. 4:**
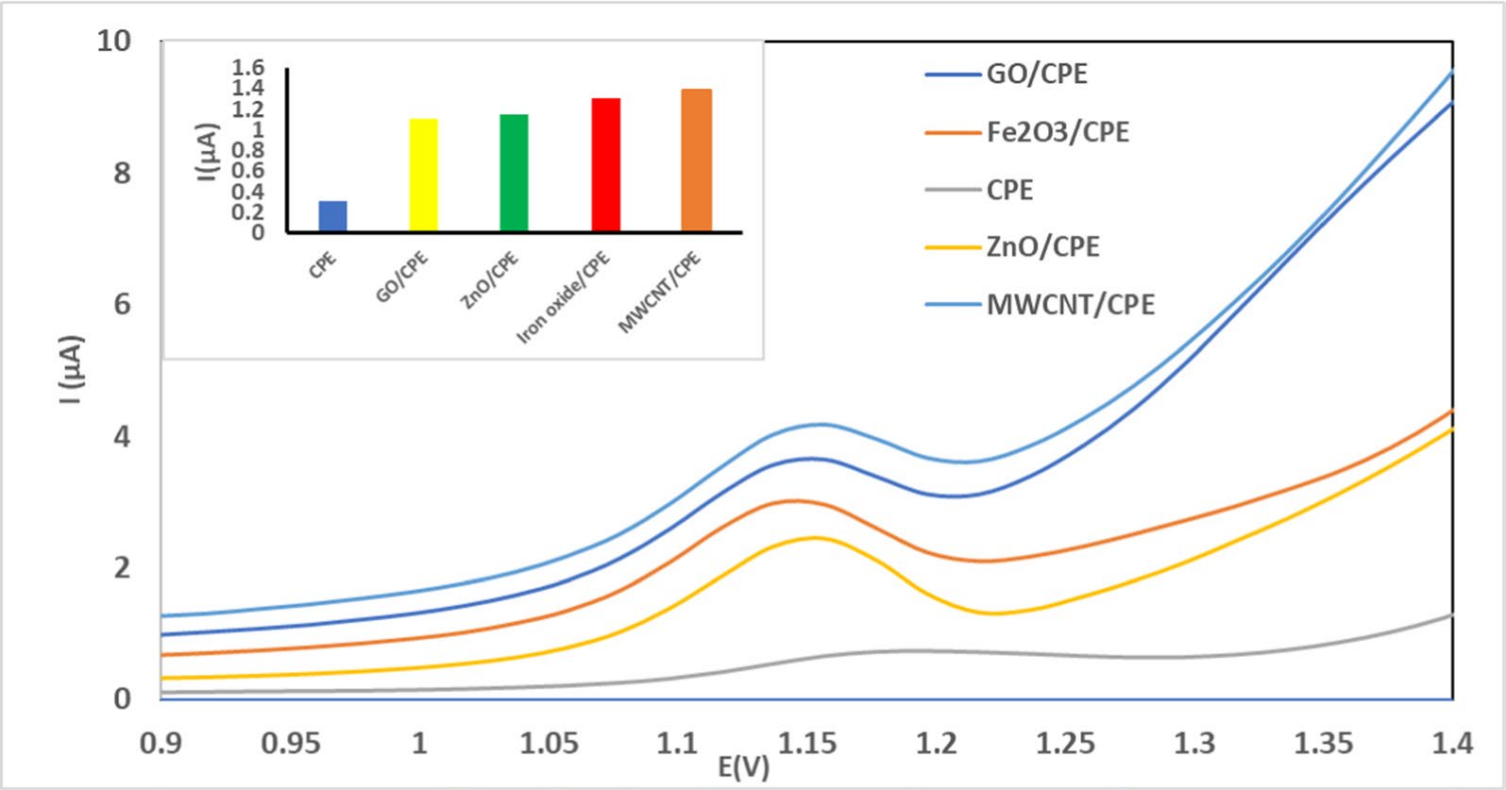
DP voltammogram of different modifications of CPE on oxidation peak height of 198.0 ng/mL (3.9 × 10^–4^ M) of TIR in 0.04 M BR buffer pH 4.0

According to Fig. 4, MWCNT/CPE represents the sharpest peak, highest current, and best peak shape of other electrodes. So, it will be used in subsequent experiments.

#### Active surface area of the electrodes

The electrodes active surface area was determined by cyclic voltammetry 1.0 mM K_4_ Fe(CN)_6_ at various scan rates using the Randles–Sevcik equation:[23]

$${\text{Ipa }} = \, \left( {{2}.{69 } \times { 1}0^{{5}} } \right){\text{ n}}^{{{3}/{2}}} \times {\text{ A}}_{0} \times {\text{ C}}_{0} \times {\text{ D}}_{0}^{{{1}/{2}}} \times \,\upsilon^{{{1}/{2}}}$$

where, **Ipa** is the current of anodic peak (μA), **A**_**0**_ is the electrode active surface area (Cm^2^), **C**_**0**_ is K_4_Fe(CN)_6_ concentration (mmol L^−1^)**, n** is the electron’s number included in the reaction, **D**_**0**_ is diffusion coefficient of the electroactive species (Cm^2^ S^−1^), and **ʋ** is scan rate (V S^−1^). For 0.1 mM K_4_Fe (CN)_6_, D_o_ = 7.63 × 10 ^−6^ cm^2^ s^−1^, n = 1.

By plotting the peak current (I_pa_) vs square root of the scan rate (*υ*^1/2^), the electroactive surface area can be calculated. The electrodes’ active surface area was measured as 0.185, 0.032, 0.131, 0.143, and 0.095 cm^2^ for MWCNT/CPE, Gr-NPs/CPE, ZnO-NPs/CPE, Fe_2_O_3_-NPs/CPE, and bare CPE, respectively as shown in Table 1., MWCNT/CPE has the largest electroactive surface area compared to the other tested electrodes.

**Table 1:** Electrochemical parameters for calculations the active surface area of various modified electrodes

Parameters	CPE	MWCNT/CPE	Gr-NPs/CPE	ZnO-NPs/CPE	Fe_2_O_3_-NPs
I_P_	2.23	4.35	0.751	3.08	3.36
Slope	7.05	13.75	2.38	9.73	10.62
Active surface area	0.095	0.185	0.032	0.131	0.143

#### Morphological and characterization of the modified electrode

**Fig. 5:**
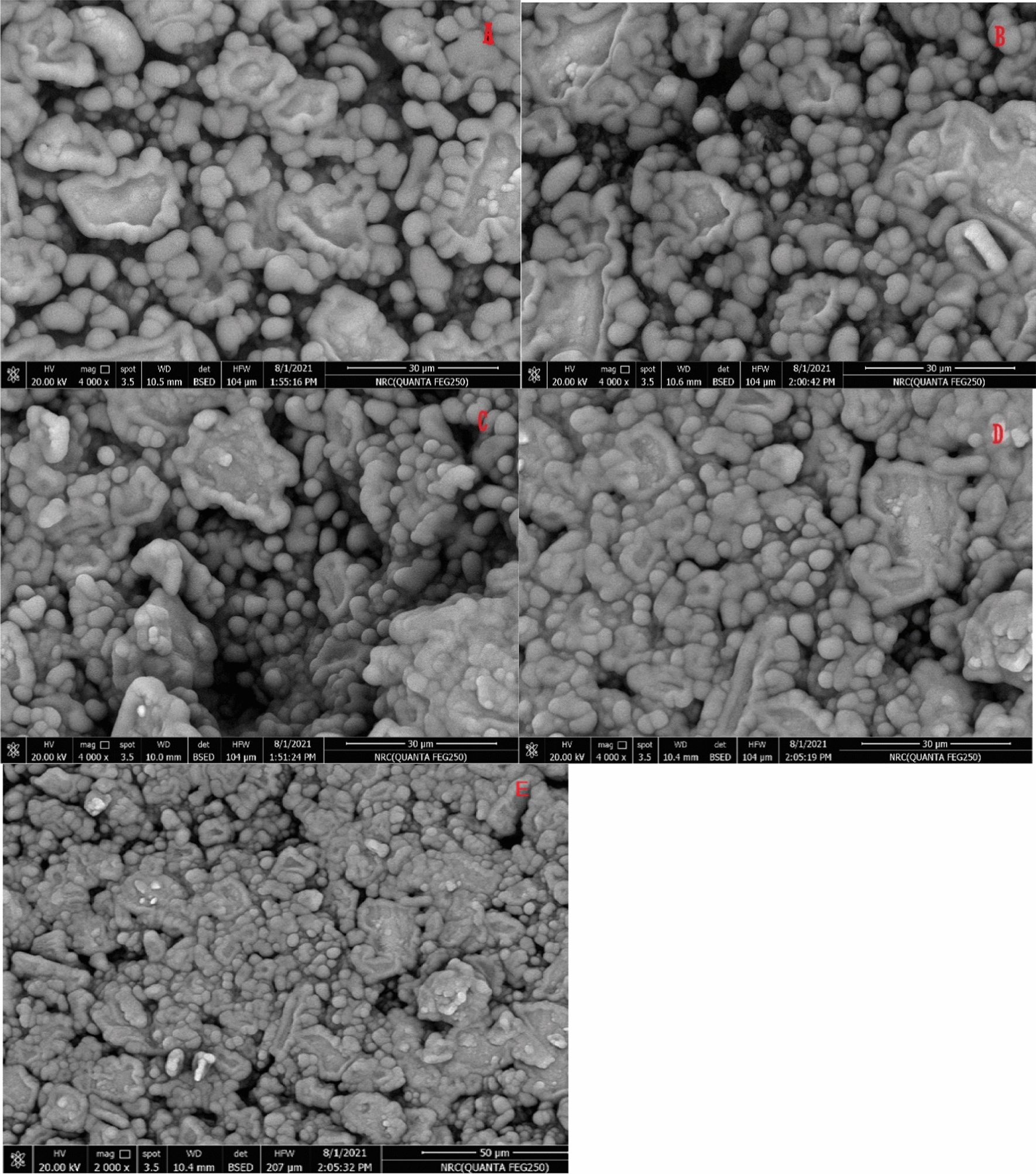
SEM images of (**A**) Fe_2_O_3_-NPs/CPE, (**B**) Gr-NPs/CPE, (**C**) MWCNT/CPE, (**D**) ZnO-NPs/CPE, and (**E**) bare CPE

**Fig. 6:**
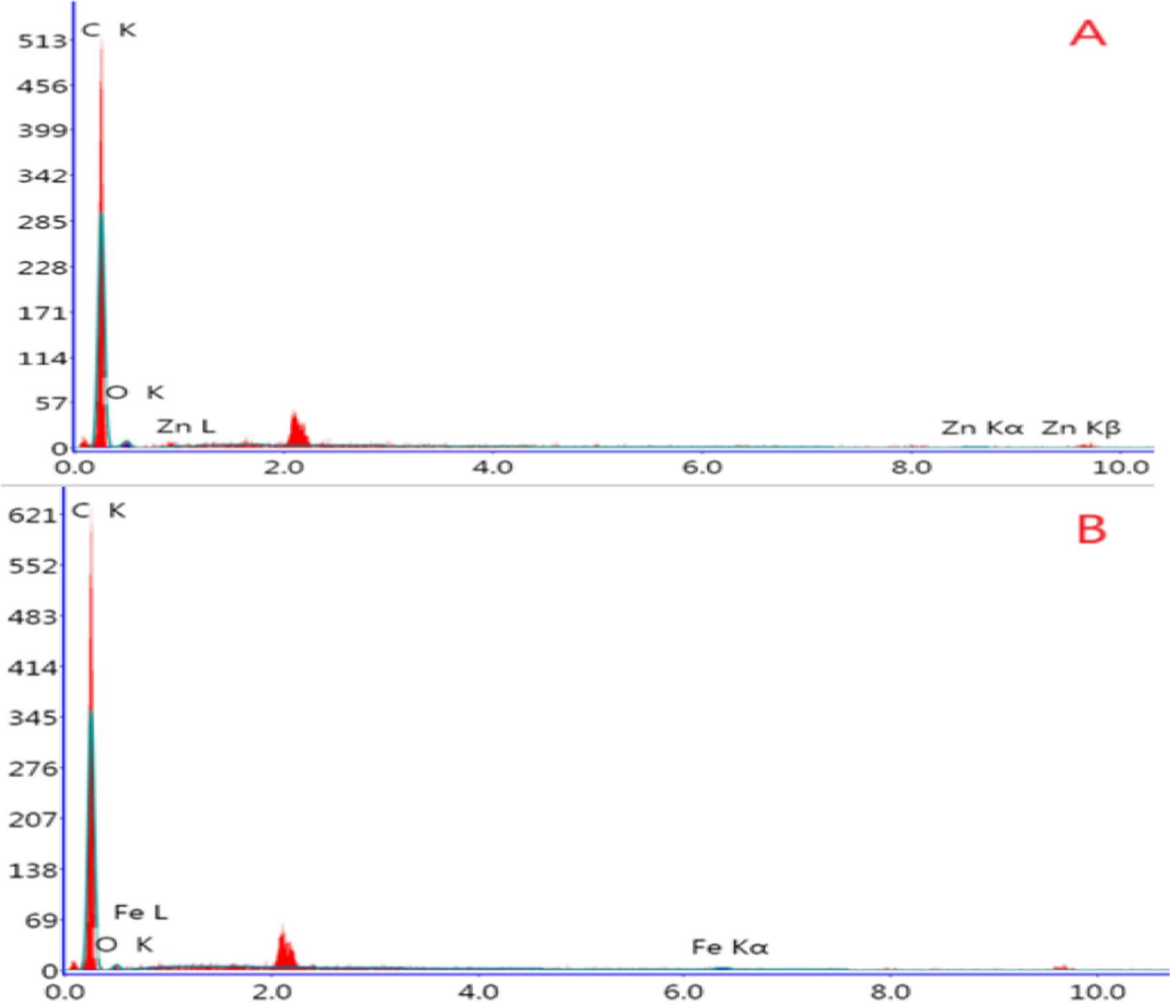
EDAX images of (**A**) ZnO-NPs/CPE and (**B**) Fe_2_O_3_-NPs/CPE

The scanning electron microscopy (SEM) technique was used to study the morphology of Fe_2_O_3_-NPs/CPE, Gr-NPs/CPE, MWCNT/CPE, ZnO-NPs/CPE, and bare CPE. As shown in Fig. 5 a compact surface of all electrodes ensures good mixing between nanoparticles, paraffin oil, and graphite powder. They also comprise a diverse array of round structures with varied dimensions. in Fig. 5C MWCNT appears uniform distribution of MWCNT and includes many big cavities and groves that look like a flower with small particle sizes increasing electroactive surface area these cavities can act as a selective adsorbent to TIR that improves electron transfer through the electrode. Energy dispersive X-ray spectroscopy (EDX) was used to support the homogeneity of the electrode matrix and confirm the content of electrodes. ZnO-NPs/CPE showed three main elements (carbon, oxygen, and zinc), and Fe_2_O_3_-NPs/CPE showed also three main elements (carbon, oxygen, and iron). The EDX spectrum reveals the C peak as the highest peak, reflecting the greater concentration of carbon present in the electrode and a weak oxygen peak due to the diffusion of air in the paste during the preparation procedure as in Fig. 6.

#### Scan rate effect

**Fig. 7:**
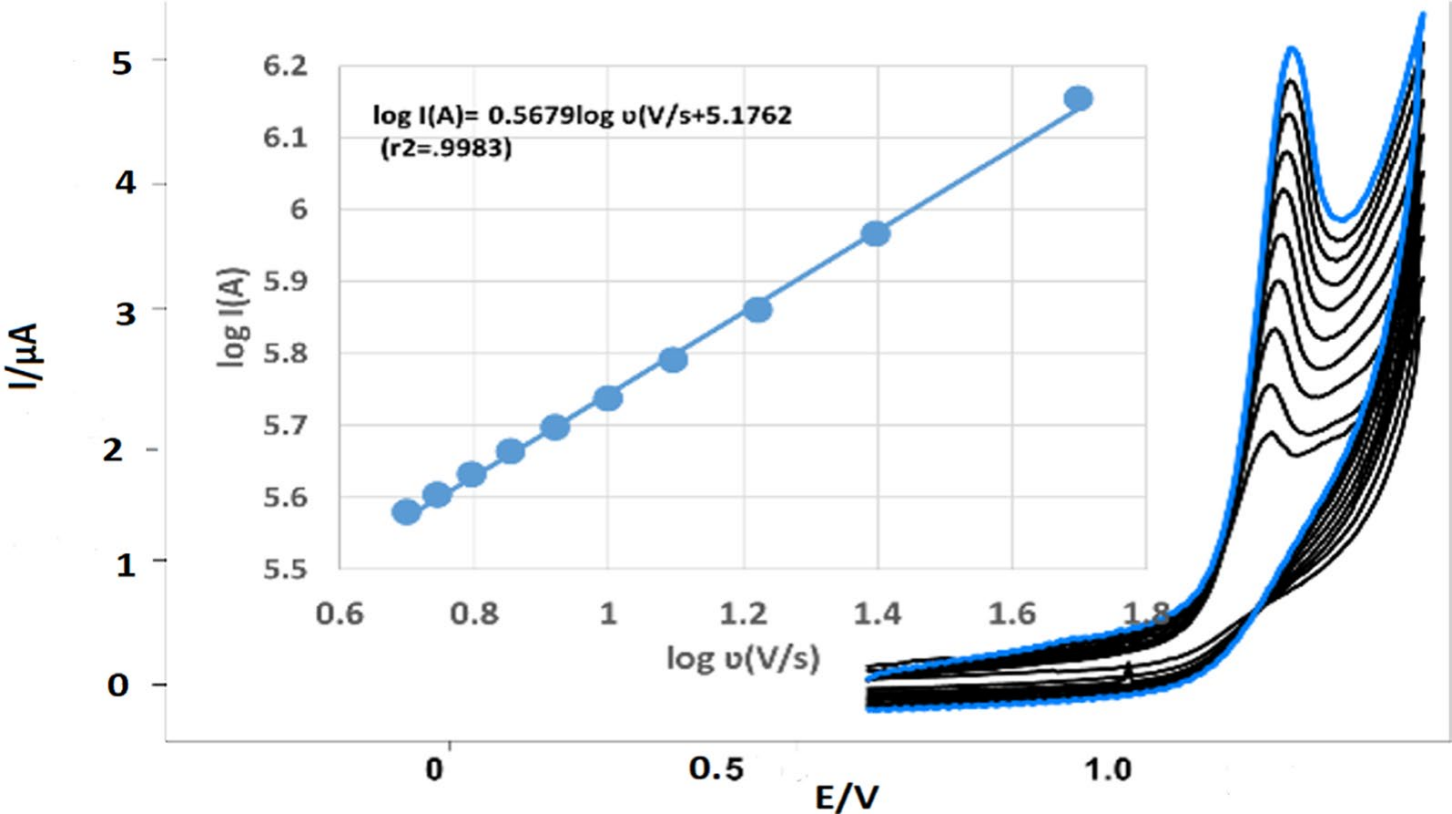
CVs of 333.00 ng/mL Tirofiban HCl at different scan rates (20–180 mV/s) in 0.04M BR-buffer at pH 4.0 on MWCNT/CPE. The inner diagram represents the plot of log I (A) vs. log ʋ

The cyclic voltammetry (CV) of TIR 0.04 M BR buffer of pH 4.0 showed no reduction peak and only one irreversible oxidation peak. By performing CV at different potential scan rates of 20–180 mV/s, a useful explanation of the electrochemical oxidation mechanism of TIR can be obtained. Linearity was obtained by plotting the logarithm of the anodic peak currents (log I_pa_) versus the logarithm of the scan rates (logʋ), with a linear regression equation of log I_pa_ = 0.5679 logʋ + 5.1762(= 0.9983), as presented in Fig. 7 the slope is 0.5679, which is very close to the theoretical slope of 0.5, indicating that the electro-oxidation was diffusion-controlled process [24]. At a scan rate of 100 mV/s, a well-defined peak shape with good repeatability was achieved, so it was chosen to proceed with method development and validation.

**Fig. 8:**
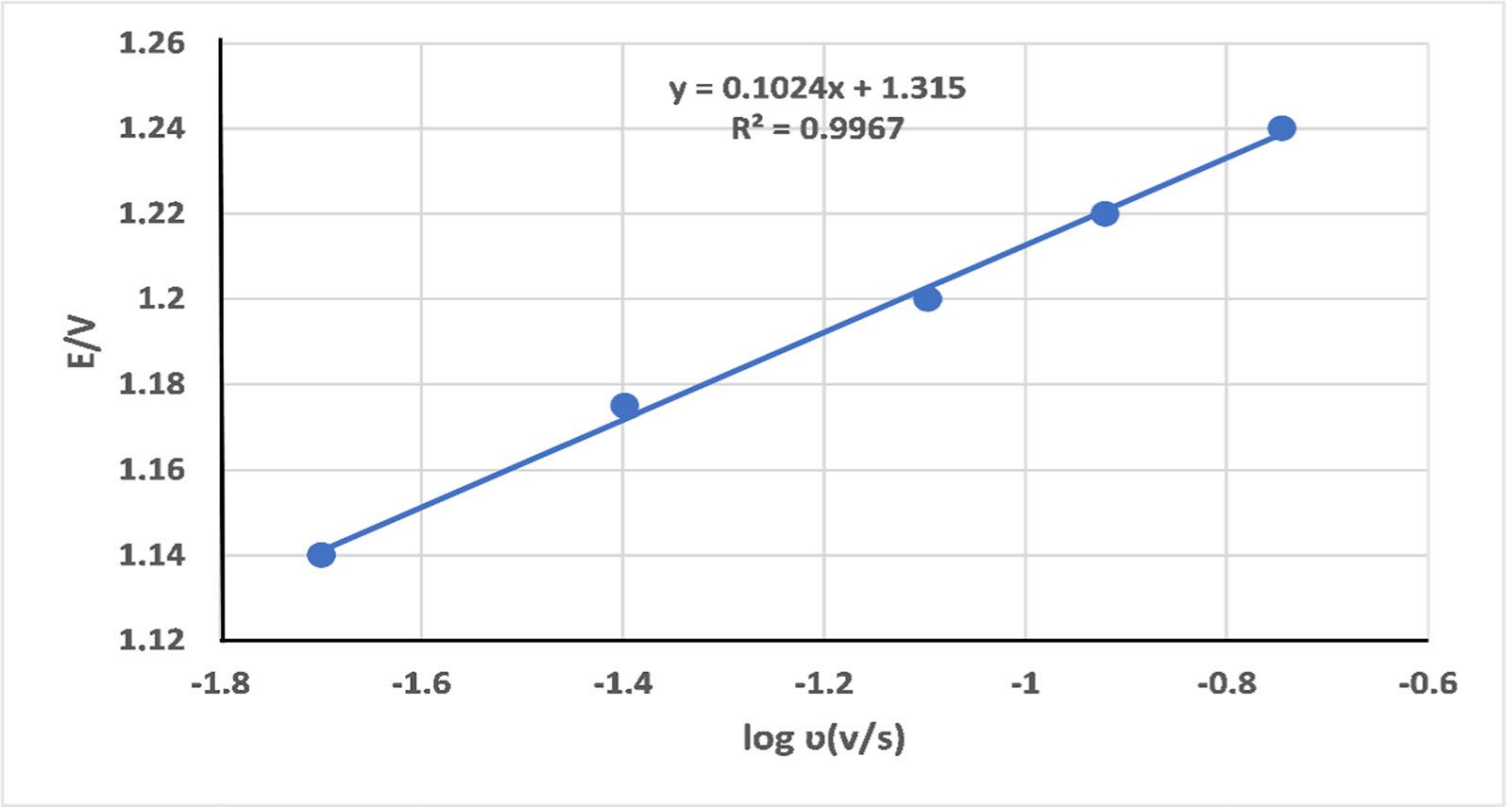
Effect of log scan rate *υ* against the potential E of TIR at MWCNT/CPE at 0.04 M BR-buffer pH 4.0

**Scheme.1:**
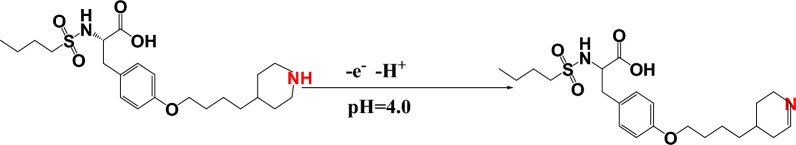
Proposed electrochemical oxidation of tirofiban HCl

The Laviron equation [25] Ep = E^0^ + 2.303RT/α n F [log RT / α n F + log ʋ] for an irreversible process can be used to calculate the number of electrons involved in the reaction, as shown below in Fig. 8. Thus, we determine αn from the slope of the relationship between E versus log v. The slope was found to be 0.1024, and generally, α (electron transfer coefficient) was assumed to be 0.5. Therefore, the number of electrons was (n = 1.28 ((≈1)), which confirms the suggested electro-oxidation mechanism of TIR as presented in Scheme 1.

#### Electrode lifetime

**Fig. 9:**
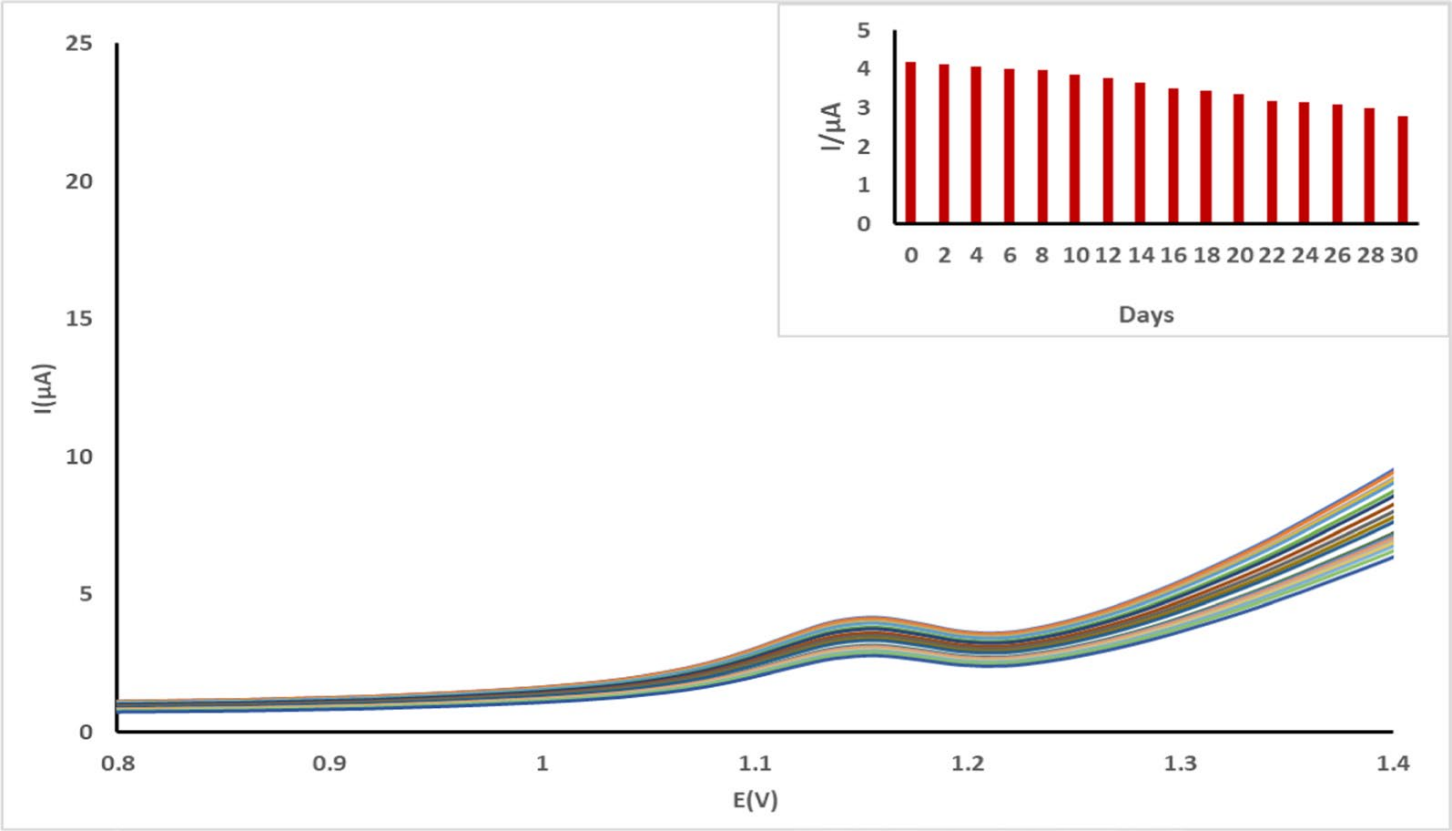
Shows the voltammogram recorded for the MWCNT/CPE electrode in 0.04 M BR buffer at pH 4.0 every couple of days for a month

The shelf life of MWCNT/CPE electrode was examined by following up the voltammogram every couple of days for a month as shown in Fig. 9. The stability of the electrode was good a long this period that confirming the good role of the MWCNT for improving the stability without losing sensitivity. The electrode was stored at a room temperature 25 ºC during this period.

### Method validation

Our electrochemical method was validated according to ICH guidelines [26]. linearity and range, the limit of detection (LOD), the limit of quantitation (LOQ), precision, and accuracy were validated.

#### Linearity and range

**Fig. 10:**
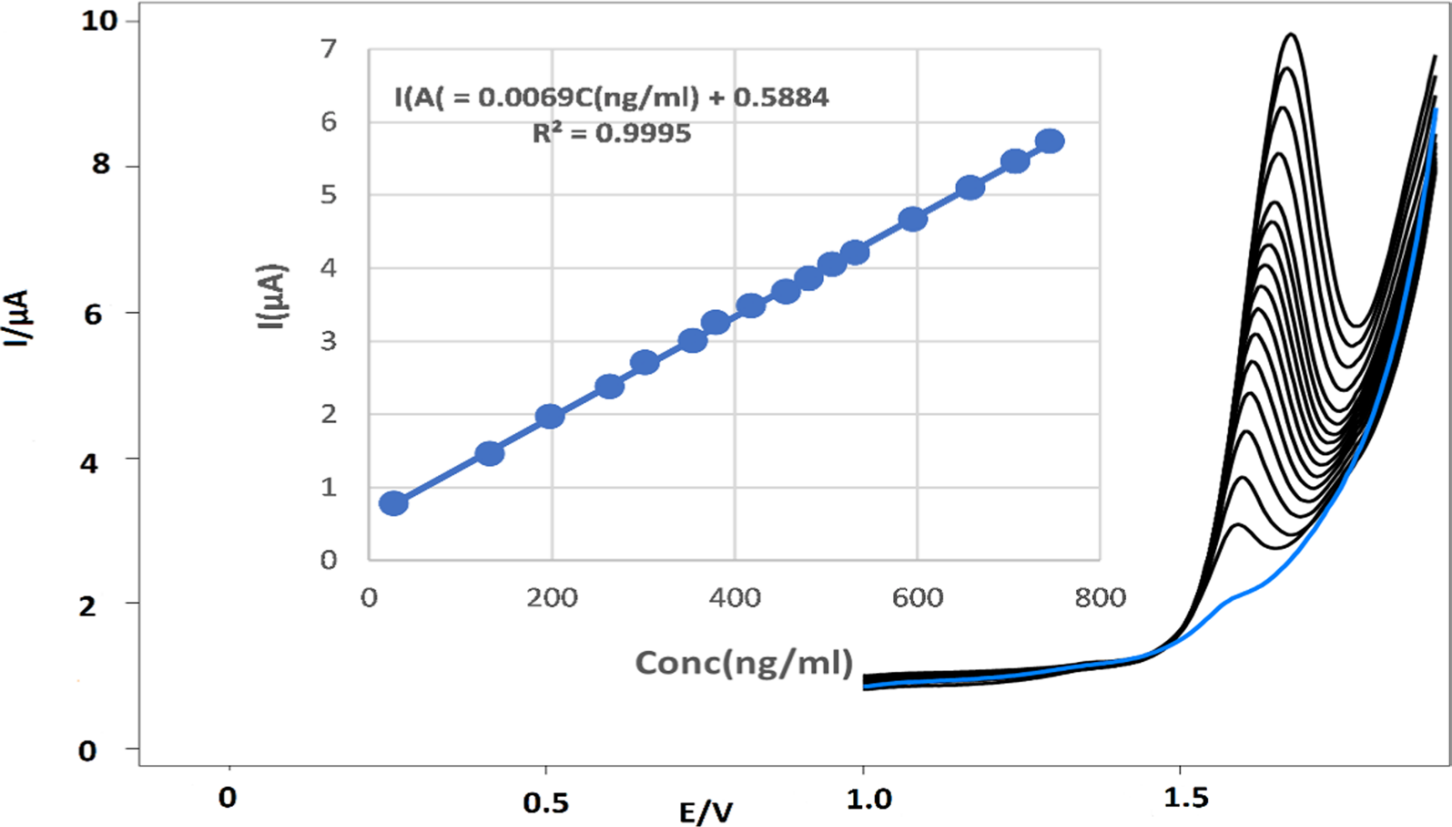
Differential pulse voltammograms and the corresponding calibration curve were recorded for different concentrations of tirofiban HCl from 27 to 745 ng mL^−1^ (5.4 × 10^–5^ M–1.5 × 10^−3^ M) using MWCNT/CPE in 0.04 M BR buffer pH 4.0 at scan rate 100 mV s^−1^

Under the optimum experimental conditions by using the DPV method the anodic peak current was directly proportional to the concentration of TIR over the concentration range of 27.00–745.00 ng/mL. Regression equation was found to be as shown in Fig. 10.

#### Limit of detection (LOD)and limit of quantification (LOQ)

Limit of detection and limit of quantification were determined experimentally by visual inspection method according to ICH guidelines and were found to be 15.50 and 26.60 ng/mL for LOD and LOQ respectively as shown in Table 2

**Table 2:** Validation data related to the suggested method for Tirofiban HCl quantification at MWCNT/CP Electrode

Parameters	(DPV)
Linear range (ng/mL)	27.00–745.00 (5.4 × 10^–5^ M-1.5 × 10^−3^ M)
Coefficient of determination (R^2^)	0.9995
Slope	0.0069
SD of slope	1.64
Intercept	0.5884
Accuracy (Mean% ± SD)	100.25 ± 1.64
Reproducibility (%RSD)	0.952
Repeatability (%RSD)	1.247
LOQ (ng/mL)	26.60 (5.4 × 10^–5^ M)
LOD (ng/mL)	15.50 (3.1 × 10^–5^ M)
S.E	0.41
%E	0.41

Where SD is the standard deviation, RSD is the relative standard deviation, S.E is the standard error, %E is the percent error

#### Accuracy

The accuracy of our suggested method can be determined by calculating the % recovery of the standard TIR. It was found to be 100.25% ± 1.64. Moreover, the accuracy of our method in pharmaceutical dosage form Table 4, was assessed by the standard addition technique. The results were obtained after three repetitions of a known concentration of the sample being spiked with a known concentration of drug standard solution.

**Table 3:** Precision data of TIR in pure form using our suggested method:

Parameters	Repeatability			Intermediate precision		
Conc (ng/mL)	302	456	595	302	456	595
Recovery %	101.81	98.26	99.42	101.33	101.81	100.37
	98.93	98.58	98.69	98.58	99.21	98.26
	100.37	100.48	101.12	98.93	98.69	98.20
Mean	100.37	99.11	99.74	101.17	98.68	98.61
± SD	1.44	1.20	1.25	0.73	0.49	0.37
RSD%	1.43	1.21	1.25	0.72	0.49	0.38
%E	0.83	0.70	0.72	0.42	0.28	0.22

**Table 4:** Application of the suggested method for determination of TIR in Clograstate dosage form:

Parameters	Added (ng/mL)	Found (ng/mL)	Bais%	Recovery (%)*	HPLC (5)
Clograstat^®^ (12.5 mg /50 mL) 200 ng/mL (4 × 10^–4^ M)	100	300.03	0.03	100.03	99.99
	300	499.95	0.017	99.98	99.97
	500	700.1	0.02	100.02	100.01
Mean*				100.02	100.00
Variance				0.000492	0.000292
SD				0.022174	0.017078
Student-t-test** (1.943)				1.429	
P				0.202884	
F-test (9.277)**				1.686	

**Values between parenthesis are the tabulated values of t and F at P = 0.05

^*^Mean of three measurements

^*^Mean of three determinations

**Table 5:** Comparison between the proposed method and the different reported methods

Method	Linear range	LOD	References
Spectrofluorimetric	0.20–5.00 µg/mL	0.019 μg/mL	[16]
HPLC/UV	0.03–0.18 µg/mL	0.00184 μg/mL	[27]
HPLC/UV	10–250 µg/mL	1.76 μg/mL	[7]
Voltammetric using CuONPs	0.06—7.41 μg/mL	20.7 ng/mL	[17]
Our proposed method using MWCNT/CPE	27.00–745.00 ng/mL	15.50 ng/mL	

#### precision

The precision of our suggested method was assessed by replicating the analysis of the TIR using three different concentrations of TIR per day to determine intra-day precision (repeatability) and on three successive days to determine inter-day precision (intermediate precision). High values of recoveries and low values of relative standard deviation %RSD prove the good precision of the proposed method Table 3.

#### Selectivity

The selectivity of our suggested method was studied in presence of other compounds that are likely to be in pharmaceutical dosage form and biological fluids such as ascorbic acid, lactose, mannitol, and sodium chloride. The interfering compounds were used at concentrations about 100 times higher than that of TIR The variation in the peak height was found to be < 3.0% for these compounds. The experimental interference results are summarized in Table 6. The procedure was done under the optimum conditions of the suggested method with a high % recovery where the interference didn’t significantly interfere with the height of the peak current of TIR.

**Table 6:** Effect of some excipients and interferants on the voltammetric response of 400 ng/L of TIR

Interferants	% signal change
Ascorbic acid	2.6
Lactose	1.1
Mannitol	1.2
Sodium chloride	0.9

### Analytical application

#### Determination of TIR in pharmaceutical dosage form

The standard addition method was used to analyze TIR in pharmaceutical dosage form for more accuracy. Excellent results were achieved (Tables 4 and 5) with low standard deviation which indicates adequate accuracy and precision of the proposed method for the application to pharmaceutical dosage form. Using the t-test and F-test, the statistical results of the suggested method were compared to those of other reported methods, confirming that there was no significant difference between them.

#### Comparative analysis

Our proposed method was compared to the methods that had previously been reported especially the voltammetric one. According to the findings presented in Table 5, the voltammetric analysis by MWCNT-CPE has superior approaches in terms of selectivity, sensitivity, and wide linear range. MWNT-CPE is distinguished by its simplicity, speed, selectivity, low cost of operation, and lack of pretreatment operations.

### Assessment of method greenness

Analytical methods use a lot of chemicals that may generate toxic residues in the environment. Herein green analytical chemistry was introduced in 2000 to remove or reduce the hazardous effect on the environment [28]. various assessment tools were introduced to evaluate the greenness of the analytical method depending on wastes, energy consumption, and solvents used per study. Each tool has a unique assessment protocol advantages and disadvantages so in this study we applied two assessment tools in green assessment as the analytical eco-scale assessment (ESA) [29] and the newest tool in a green assessment called complex green analytical procedure index (Complex-GAPI). ESA has the advantage of providing a quantitative evaluation of the analytical methods by considering all of the reagents used, rather than just the most hazardous ones, as other matrices do. It is based on penalty points subtracted from a 100-mark total eco scale score if more than 75 represents excellent greenness, more than 50 represents acceptable greenness and less than 50 represents inadequate greenness Table 7 represents the scores for the suggested method. Our method achieved 77 penalty points, which demonstrated its excellent greenness. It has several disadvantages including insufficient information about the causes of the analytical procedure’s environmental impact and no data about the structure of the hazards is founded. Our method is regarded as an excellent green analysis.

**Table 7:** Green assessment of the suggested method by two approaches eco-scale and complex-gapi

	Eco-scale parameter	Penalty points	Diagram of complex-gapi
reagents	Boric acid Graphite Glacial acetic acid Paraffin oil Phosphoric acid Methanol Sodium hydroxide	2 1 4 2 2 6 2	
instrument	Voltameter pH meter Vortex mixer Centrifuge Occupational hazard Waste (10–100 mL, degradation)	0 0 0 0 0 4	
∑penalty		23	
Total score		77	

Complex-GAPI is the most recent advanced technique or assessment of green chemistry. It is the developed version of the GAPI assessment. It represents a comprehension evaluation of the whole analytical process from sample collection to final analysis including transport, preservation, storage, and sample preparation. The scale is based on utilizing 5 pentagrams and the additional hexagonal part is the pre-analysis step. The green color represents an eco-friendly step yellow color represents a medium environmental impact and the red color represents a hazardous environmental impact of this step [30]. There is no need for a purification step, which corresponds to the white region in the hexagonal shape. This technique has a lot of advantages as the software is available that will facilitate the use of such a tool, it is simple, friendly use, and includes all the factors that characterize the analytical protocol as well as the pre-analysis process (conditions, techniques, and reagents).

## Conclusion

This work has been represented as the first successful green method for investigating voltammetric behavior and determining TIR in pure form and pharmaceutical dosage form by MWCNT/CPE electrode in B-R buffer pH 4.0. The suggested method provides more information about the mechanism of electrochemical oxidation of TIR and shows good accuracy, sensitivity, selectivity, and precision. This method is also eco-friendly and suitable for use in quality control laboratories with the elimination of carcinogenic and hazardous chemicals.

## Data Availability

The datasets used and/or analysed during the current study are available from the corresponding author on reasonable request.

## Abbreviations

TIR: Tirofiban hydrochloride
CPE: Carbon paste electrode
DPV: Differential pulse voltammetry
MWCNT: Multi-wall carbon nanotube
Gr: Graphene
ESA: Analytical eco-scale assessment
Complex-GAPI: Complex Green Analytical Procedure Index
